# Imidazolium *trans*-bis­(imino­diacetato-κ^3^ *O*,*N*,*O*′)cobaltate(III)

**DOI:** 10.1107/S1600536809014342

**Published:** 2009-04-25

**Authors:** Xiao-Li Gao, Li-Ping Lu, Miao-Li Zhu

**Affiliations:** a Institute of Molecular Science, Key Laboratory of Chemical Biology and Molecular Engineering of the Education Ministry, Shanxi University, Taiyuan, Shanxi 030006, People’s Republic of China

## Abstract

In the title compound, (C_3_H_5_N_2_)[Co(C_4_H_5_NO_4_)_2_], the cation and anion are located on a twofold rotation axis and inversion center, respectively. Inter­molecular N—H⋯O hydrogen bonds link the cations and anions into layers parallel to the *ab* plane. The crystal packing also exhibits weak C—H⋯O hydrogen bonds, including bifurcated hydrogen bonds, and C=O⋯π inter­actions.

## Related literature

For hydrogen bonds in related compounds containing imidazolium, see: Allen (2002 ▶); Chattopadhyay *et al.* (1995 ▶); Gao *et al.* (2009 ▶); Hsu & Schlemper (1980 ▶); Rissanen & Pursiainen (2000 ▶). Bifurcated hydrogen bonds were discussed by Jeffrey *et al.* (1985 ▶). For graph-set notation, see: Bernstein *et al.* (1995 ▶).
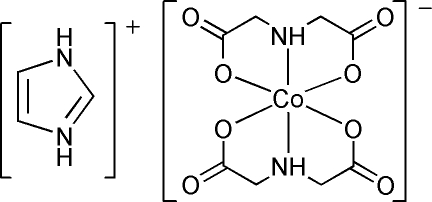


## Experimental

### 

#### Crystal data

(C_3_H_5_N_2_)[Co(C_4_H_5_NO_4_)_2_]
*M* *_r_* = 390.20
Orthorhombic, 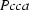

*a* = 16.889 (3) Å
*b* = 5.2906 (10) Å
*c* = 16.901 (3) Å
*V* = 1510.2 (5) Å^3^
*Z* = 4
Mo *K*α radiation
μ = 1.19 mm^−1^
*T* = 298 K
0.20 × 0.20 × 0.20 mm

#### Data collection

Bruker SMART CCD area-detector diffractometer
Absorption correction: multi-scan (*SADABS*; Sheldrick, 2000 ▶) *T*_min_ = 0.783, *T*_max_ = 0.797
6218 measured reflections
1495 independent reflections
1299 reflections with *I* > 2σ(*I*)
*R*_int_ = 0.025

#### Refinement

*R*[*F* ^2^ > 2σ(*F* ^2^)] = 0.029
*wR*(*F* ^2^) = 0.079
*S* = 1.08
1495 reflections
111 parameters
H-atom parameters constrained
Δρ_max_ = 0.35 e Å^−3^
Δρ_min_ = −0.27 e Å^−3^

### 

Data collection: *SMART* (Bruker, 2000 ▶); cell refinement: *SAINT* (Bruker, 2000 ▶); data reduction: *SAINT*; program(s) used to solve structure: *SHELXS97* (Sheldrick, 2008 ▶); program(s) used to refine structure: *SHELXL97* (Sheldrick, 2008 ▶); molecular graphics: *SHELXTL/PC* (Sheldrick, 2008 ▶); software used to prepare material for publication: *SHELXTL/PC*.

## Supplementary Material

Crystal structure: contains datablocks I, global. DOI: 10.1107/S1600536809014342/cv2551sup1.cif

Structure factors: contains datablocks I. DOI: 10.1107/S1600536809014342/cv2551Isup2.hkl

Additional supplementary materials:  crystallographic information; 3D view; checkCIF report

## Figures and Tables

**Table 1:** Hydrogen-bond geometry (Å, °) *Cg*1 is the centroid of the imidazolium ring.

*D*—H⋯*A*	*D*—H	H⋯*A*	*D*⋯*A*	*D*—H⋯*A*
N1—H1⋯O3^i^	0.79	2.12	2.880 (2)	161
N2—H2⋯O2^ii^	0.86	1.95	2.775 (3)	162
C3—H3*B*⋯O2^iii^	0.97	2.43	3.338 (3)	156
C5—H5⋯O4^i^	0.93	2.36	3.117 (4)	138
C5—H5⋯O4^iv^	0.93	2.36	3.117 (4)	138
C1—O2⋯*Cg*1^v^	1.23 (1)	3.54 (1)	3.953 (2)	0 ?

Symmetry codes: (i) 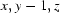; (ii) 
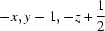
; (iii) 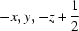; (iv) 
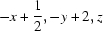
; (v) 
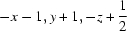
.

**Fig. 1.**
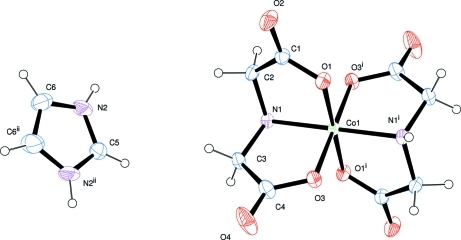
View of (I) with displacement ellipsoids drawn at the 30% probability level and atomic numbering [symmetry codes: (i) -x, -y, -z; (ii) 1/2 - x, -1 - y, z].

**Fig. 2.**
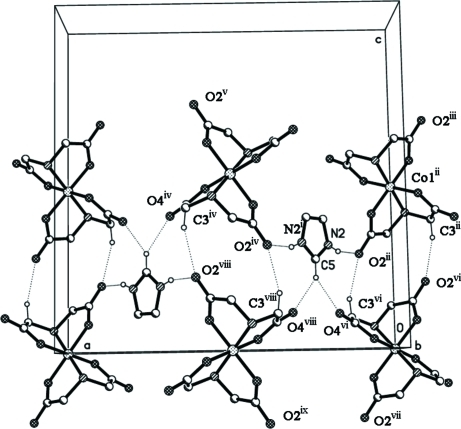
A portion of the crystal packing showing hydrogen-bonding patterns with graph-set notations R32(9), R32(9) and R22(12), respectively. Dotted lines denote hydrogen bonds. H atoms non-involved in hydrogen-bonding omitted for clarity. [Symmetry codes: (i) 1/2 - x, 1 - y, z; (ii) -x, -1 + y, 1/2 - z; (iii) x, 1 - y, 1/2 + z; (iv) 1/2 + x, 2 - y, 1/2 - z; (v) 1/2 - x, y, 1/2 + z; (vi) x, -1 + y, z; (vii) -x, 1 - y, -z; (viii) 1/2 - x, 2 - y, z; (ix) 1/2 + x, y, -z.]

## supplementary crystallographic information

### Comment

In continuation of our study of weak C—H···O and C═O···π interactions in the building of three-dimensional structures (Gao *et al.*, 2009), we present here the crystal structure of the title 1:1 adduct, (I).

In (I) (Fig. 1), the cation and anion are located on a two-fold rotation axis and inversion center, respectively. Strong N2—H···O2^ii^  hydrogen bonds (Table 1) link imidazolium and *trans*-bis(iminodiacetato-N,*O*,*O*')cobalt(III) with the direction of (120) to form one-dimensional chains (Fig. 2) with graph-set notation C_2_^2^(13). These chains further assemble to other *trans*-bis(iminodiacetato-N,*O*,*O*')cobalt(III) anions in two-dimensional structures on (001) plane *via* C5—H···O4^vi^, C5—H···O4^viii^ and C3^viii^—H···O2^iv^ (Table 1) hydrogen bonds, as shown in Fig. 2. Further, the layers assemble to a three-dimensional structure by strong N1—H···O^i^ hydrogen bond and weak C1═O2···π(centroid of imidazolium ring) interaction (Table 1). Imidazolium has three donors, two N—H and one C—H, the latter forming a non-conventional bifurcated hydrogen bond between imidazolium C5—H and carboxylate groups O4H and O4F from *trans*-bis(iminodiacetato-N,*O*,*O*')cobalt(III). Interestingly, this type of hydrogen bond in imidazolium compounds was found in a search of the Cambridge Structural Database (CSD version 5.30; Allen, 2002). Among the 200 hits for imidazolium compounds, there are only seven compounds having the similar bifurcated hydrogen bonds (C—H from imidazolium), namely, imidazolium hydrogen maleate (Hsu & Schlemper, 1980), benzo-18-crown-6 imidazolium clathrate perchlorate (Rissanen & Pursiainen, 2000), bis(imidazolium) bis(oxalato-O,*O*')copper(II) (Chattopadhyay *et al.*, 1995). Their distances of H···O are in the range of 2.10 to 2.49 Å. However, analysis in sum of three angles about H atom are all less than 360° in the seven compounds, which indicated that these weak hydrogen bonds are not of characters of bifurcated hydrogen bonds from H-bond classification (Jeffrey *et al.*, 1985).

### Experimental

Chemicals were readily available from commercial sources and were used as received without further purification. To a 10 ml of solution containing imidazole (0.14 g, 2 mmol) in a flask with constant stirring, added dropwise Co(CH_3_COO)_2_.3H_2_O (0.23 g, 1 mmol) in 5 ml of aqueous solution and 5 ml aqueous solution containing iminodiacetic acid (0.27 g, 2 mmol) was added dropwise. The mixture was stirred for 3 h, and then filtered. The dark-red filtrate was left to stand at room temperature, and after three weeks, dark-red crystals of the title compound were formed.

### Refinement

H atoms attached to C atoms of (I) were placed in geometrically idealized positions and refined with *U*_iso_(H)=1.2*U*_eq_(C). H atoms attached to N1 and N2 in (I) were located from difference Fourier maps, with fixed bond lengths, and refined using a riding model, with *U*_iso_(H) = 1.2*U*_eq_(N).

### Crystal data

**Table:**

(C_3_H_5_N_2_)[Co(C_4_H_5_NO_4_)_2_]	*F*(000) = 800
*M**_r_* = 390.20	*D*_x_ = 1.716 Mg m^−^^3^
Orthorhombic, *P**c**c**a*	Mo *K*α radiation, λ = 0.71073 Å
Hall symbol: -P 2a 2ac	Cell parameters from 2958 reflections
*a* = 16.889 (3) Å	θ = 2.4–27.0°
*b* = 5.2906 (10) Å	µ = 1.19 mm^−^^1^
*c* = 16.901 (3) Å	*T* = 298 K
*V* = 1510.2 (5) Å^3^	Block, red
*Z* = 4	0.20 × 0.20 × 0.20 mm

### Data collection

**Table:**

Bruker SMART CCD area-detector diffractometer	1495 independent reflections
Radiation source: fine-focus sealed tube	1299 reflections with *I* > 2σ(*I*)
graphite	*R*_int_ = 0.025
ω scans	θ_max_ = 26.0°, θ_min_ = 2.4°
Absorption correction: multi-scan (*SADABS*; Sheldrick, 2000)	*h* = −19→20
*T*_min_ = 0.783, *T*_max_ = 0.797	*k* = −6→4
6218 measured reflections	*l* = −18→20

### Refinement

**Table:**

Refinement on *F*^2^	Primary atom site location: structure-invariant direct methods
Least-squares matrix: full	Secondary atom site location: difference Fourier map
*R*[*F*^2^ > 2σ(*F*^2^)] = 0.029	Hydrogen site location: inferred from neighbouring sites
*wR*(*F*^2^) = 0.079	H-atom parameters constrained
*S* = 1.08	*w* = 1/[σ^2^(*F*_o_^2^) + (0.0405*P*)^2^ + 0.6086*P*] where *P* = (*F*_o_^2^ + 2*F*_c_^2^)/3
1495 reflections	(Δ/σ)_max_ < 0.001
111 parameters	Δρ_max_ = 0.35 e Å^−^^3^
0 restraints	Δρ_min_ = −0.27 e Å^−^^3^

### Special details

**Table:**

Geometry. All e.s.d.'s (except the e.s.d. in the dihedral angle between two l.s. planes) are estimated using the full covariance matrix. The cell e.s.d.'s are taken into account individually in the estimation of e.s.d.'s in distances, angles and torsion angles; correlations between e.s.d.'s in cell parameters are only used when they are defined by crystal symmetry. An approximate (isotropic) treatment of cell e.s.d.'s is used for estimating e.s.d.'s involving l.s. planes.
Refinement. Refinement of *F*^2^ against ALL reflections. The weighted *R*-factor *wR* and goodness of fit *S* are based on *F*^2^, conventional *R*-factors *R* are based on *F*, with *F* set to zero for negative *F*^2^. The threshold expression of *F*^2^ > σ(*F*^2^) is used only for calculating *R*-factors(gt) *etc*. and is not relevant to the choice of reflections for refinement. *R*-factors based on *F*^2^ are statistically about twice as large as those based on *F*, and *R*- factors based on ALL data will be even larger.

### Fractional atomic coordinates and isotropic or equivalent isotropic displacement parameters (Å^2^)

**Table:**

	*x*	*y*	*z*	*U*_iso_*/*U*_eq_	
Co1	0.0000	1.0000	0.0000	0.02108 (14)	
O1	−0.06498 (8)	1.0888 (2)	0.08656 (8)	0.0305 (3)	
O2	−0.10143 (10)	0.9556 (3)	0.20572 (10)	0.0502 (5)	
O3	0.07296 (7)	1.2495 (2)	0.03073 (9)	0.0306 (3)	
O4	0.17666 (11)	1.3151 (3)	0.10704 (14)	0.0775 (7)	
N1	0.05329 (8)	0.7739 (3)	0.07205 (9)	0.0240 (3)	
H1	0.0644	0.6440	0.0521	0.029*	
C1	−0.06105 (12)	0.9354 (4)	0.14545 (12)	0.0319 (4)	
C2	−0.00355 (11)	0.7182 (4)	0.13637 (12)	0.0315 (5)	
H2A	0.0249	0.6926	0.1856	0.038*	
H2B	−0.0324	0.5644	0.1242	0.038*	
C3	0.12727 (11)	0.8972 (4)	0.09931 (13)	0.0327 (4)	
H3A	0.1724	0.8161	0.0744	0.039*	
H3B	0.1324	0.8764	0.1561	0.039*	
C4	0.12754 (12)	1.1751 (4)	0.07933 (13)	0.0369 (5)	
N2	0.20741 (11)	0.3528 (4)	0.31139 (13)	0.0526 (5)	
H2	0.1749	0.2389	0.2952	0.063*	
C5	0.2500	0.5000	0.2661 (2)	0.0461 (9)	
H5	0.2500	0.5000	0.2110	0.055*	
C6	0.22287 (19)	0.4090 (8)	0.38741 (18)	0.0811 (11)	
H6	0.1999	0.3346	0.4317	0.097*	

### Atomic displacement parameters (Å^2^)

**Table:**

	*U*^11^	*U*^22^	*U*^33^	*U*^12^	*U*^13^	*U*^23^
Co1	0.0249 (2)	0.0181 (2)	0.0202 (2)	0.00274 (13)	−0.00159 (13)	−0.00175 (12)
O1	0.0359 (7)	0.0297 (7)	0.0259 (7)	0.0101 (6)	0.0047 (6)	−0.0001 (6)
O2	0.0550 (10)	0.0629 (11)	0.0325 (9)	0.0263 (8)	0.0165 (8)	0.0112 (8)
O3	0.0323 (7)	0.0210 (6)	0.0385 (8)	−0.0005 (5)	−0.0086 (6)	−0.0013 (6)
O4	0.0655 (12)	0.0340 (9)	0.1330 (19)	−0.0079 (9)	−0.0602 (12)	−0.0013 (10)
N1	0.0288 (8)	0.0187 (7)	0.0246 (8)	0.0051 (6)	−0.0019 (6)	−0.0030 (6)
C1	0.0334 (10)	0.0351 (10)	0.0271 (11)	0.0056 (9)	0.0021 (9)	0.0002 (8)
C2	0.0376 (11)	0.0297 (10)	0.0273 (11)	0.0068 (8)	0.0025 (8)	0.0064 (8)
C3	0.0301 (10)	0.0306 (10)	0.0374 (11)	0.0032 (8)	−0.0084 (9)	−0.0001 (9)
C4	0.0345 (10)	0.0269 (10)	0.0492 (13)	0.0013 (8)	−0.0115 (9)	−0.0061 (9)
N2	0.0418 (11)	0.0547 (13)	0.0613 (14)	−0.0206 (10)	−0.0043 (10)	−0.0035 (10)
C5	0.0403 (19)	0.057 (2)	0.041 (2)	−0.0004 (15)	0.000	0.000
C6	0.074 (2)	0.122 (3)	0.0467 (18)	−0.048 (2)	0.0007 (15)	0.0132 (18)

### Geometric parameters (Å, °)

**Table:**

Co1—O3^i^	1.8790 (12)	C1—C2	1.512 (3)
Co1—O3	1.8790 (12)	C2—H2A	0.9700
Co1—O1	1.8882 (13)	C2—H2B	0.9700
Co1—O1^i^	1.8882 (13)	C3—C4	1.508 (3)
Co1—N1^i^	1.9299 (14)	C3—H3A	0.9700
Co1—N1	1.9299 (14)	C3—H3B	0.9700
O1—C1	1.286 (2)	N2—C5	1.308 (3)
O2—C1	1.230 (2)	N2—C6	1.344 (4)
O3—C4	1.296 (2)	N2—H2	0.8599
O4—C4	1.207 (3)	C5—N2^ii^	1.308 (3)
N1—C2	1.480 (2)	C5—H5	0.9300
N1—C3	1.483 (2)	C6—C6^ii^	1.329 (6)
N1—H1	0.7879	C6—H6	0.9300
			
O3^i^—Co1—O3	180.00 (9)	O1—C1—C2	115.72 (17)
O3^i^—Co1—O1	90.44 (6)	N1—C2—C1	109.88 (16)
O3—Co1—O1	89.56 (6)	N1—C2—H2A	109.7
O3^i^—Co1—O1^i^	89.56 (6)	C1—C2—H2A	109.7
O3—Co1—O1^i^	90.44 (6)	N1—C2—H2B	109.7
O1—Co1—O1^i^	180.0	C1—C2—H2B	109.7
O3^i^—Co1—N1^i^	87.43 (6)	H2A—C2—H2B	108.2
O3—Co1—N1^i^	92.57 (6)	N1—C3—C4	111.22 (15)
O1—Co1—N1^i^	93.65 (6)	N1—C3—H3A	109.4
O1^i^—Co1—N1^i^	86.35 (6)	C4—C3—H3A	109.4
O3^i^—Co1—N1	92.57 (6)	N1—C3—H3B	109.4
O3—Co1—N1	87.43 (6)	C4—C3—H3B	109.4
O1—Co1—N1	86.35 (6)	H3A—C3—H3B	108.0
O1^i^—Co1—N1	93.65 (6)	O4—C4—O3	123.3 (2)
N1^i^—Co1—N1	180.00 (7)	O4—C4—C3	120.91 (19)
C1—O1—Co1	114.38 (12)	O3—C4—C3	115.83 (16)
C4—O3—Co1	115.36 (12)	C5—N2—C6	108.8 (2)
C2—N1—C3	113.95 (15)	C5—N2—H2	125.6
C2—N1—Co1	106.52 (11)	C6—N2—H2	125.6
C3—N1—Co1	108.42 (11)	N2^ii^—C5—N2	108.3 (3)
C2—N1—H1	107.2	N2^ii^—C5—H5	125.9
C3—N1—H1	108.4	N2—C5—H5	125.9
Co1—N1—H1	112.5	C6^ii^—C6—N2	107.09 (15)
O2—C1—O1	123.87 (19)	C6^ii^—C6—H6	126.5
O2—C1—C2	120.40 (18)	N2—C6—H6	126.5
			
O3^i^—Co1—O1—C1	77.41 (15)	O1^i^—Co1—N1—C3	80.29 (12)
O3—Co1—O1—C1	−102.59 (15)	Co1—O1—C1—O2	−176.80 (17)
N1^i^—Co1—O1—C1	164.87 (14)	Co1—O1—C1—C2	2.0 (2)
N1—Co1—O1—C1	−15.13 (14)	C3—N1—C2—C1	92.47 (19)
O1—Co1—O3—C4	90.08 (15)	Co1—N1—C2—C1	−27.03 (19)
O1^i^—Co1—O3—C4	−89.92 (15)	O2—C1—C2—N1	−163.64 (19)
N1^i^—Co1—O3—C4	−176.29 (15)	O1—C1—C2—N1	17.5 (3)
N1—Co1—O3—C4	3.71 (15)	C2—N1—C3—C4	−104.50 (19)
O3^i^—Co1—N1—C2	−66.96 (12)	Co1—N1—C3—C4	13.92 (19)
O3—Co1—N1—C2	113.04 (12)	Co1—O3—C4—O4	−176.8 (2)
O1—Co1—N1—C2	23.32 (12)	Co1—O3—C4—C3	3.9 (2)
O1^i^—Co1—N1—C2	−156.68 (12)	N1—C3—C4—O4	168.6 (2)
O3^i^—Co1—N1—C3	170.01 (12)	N1—C3—C4—O3	−12.2 (3)
O3—Co1—N1—C3	−9.99 (12)	C6—N2—C5—N2^ii^	0.4 (2)
O1—Co1—N1—C3	−99.71 (12)	C5—N2—C6—C6^ii^	−1.2 (5)

Symmetry codes: (i) −*x*, −*y*+2, −*z*; (ii) −*x*+1/2, −*y*+1, *z*.

### Hydrogen-bond geometry (Å, °)

**Table:**

*D*—H···*A*	*D*—H	H···*A*	*D*···*A*	*D*—H···*A*
N1—H1···O3^iii^	0.79	2.12	2.880 (2)	161
N2—H2···O2^iv^	0.86	1.95	2.775 (3)	162
C3—H3B···O2^v^	0.97	2.43	3.338 (3)	156
C5—H5···O4^iii^	0.93	2.36	3.117 (4)	138
C5—H5···O4^vi^	0.93	2.36	3.117 (4)	138
C1—O2···Cg1^vii^	1.230 (2)	3.54 (1)	3.953 (2)	.

Symmetry codes: (iii) *x*, *y*−1, *z*; (iv) −*x*, *y*−1, −*z*+1/2; (v) −*x*, *y*, −*z*+1/2; (vi) −*x*+1/2, −*y*+2, *z*; (vii) −*x*−1, *y*+1, −*z*+1/2.
